# Pharmacokinetics of Budesonide Oral Suspension in Children and Adolescents With Eosinophilic Esophagitis

**DOI:** 10.1097/MPG.0000000000003482

**Published:** 2022-06-06

**Authors:** Sandeep K. Gupta, Malcolm Hill, Joanne M. Vitanza, Robert H. Farber, Nirav K. Desai, James Williams, Ivy H. Song

**Affiliations:** From the * Section of Pediatric Gastroenterology, Hepatology and Nutrition, Indiana University School of Medicine, Riley Hospital for Children at Indiana University Health, Indianapolis, IN; the † Meritage Pharma, Inc., a Takeda company, San Diego, CA; the ‡ Takeda Development Center Americas, Inc., Cambridge, MA; the § Takeda Development Center Americas, Inc., Lexington, MA.

**Keywords:** budesonide, pediatric study, systemic pharmacokinetics, topical swallowed corticosteroid

## Abstract

The pharmacokinetic (PK) profile of budesonide oral suspension (BOS) was evaluated during a phase 2, randomized, double-blind, placebo-controlled, dose-ranging study in pediatric patients with eosinophilic esophagitis (EoE) (MPI 101-01/NCT00762073).

Non-compartmental methods were used to calculate PK parameters in 37 patients after receiving morning doses of BOS, with volume and dose adjusted for age (low dose: 0.35 or 0.5 mg; high dose: 1.4 or 2.0 mg [2–9 or 10–18 years old, respectively]). Relationships between apparent oral clearance and volume of distribution, and bodyweight and body mass index were also evaluated.

Budesonide systemic exposure increased with BOS dose. After oral administration, time to maximum plasma budesonide concentration occurred ~1 hour post dose and the half-life of budesonide was 3.3–3.5 hours. PK parameters were similar between age groups for low- and high-dose BOS, indicating that volume and dose adjustments for age were appropriate for pediatric patients with EoE. BOS was well tolerated.

**What Is Known**

- The efficacy and safety of budesonide oral suspension (BOS) for the treatment of eosinophilic esophagitis (EoE) in adult, adolescent, and pediatric patients have been described in 2 phase 2 and 2 phase 3 multicenter, randomized, placebo-controlled trials.

**What Is New**

- Budesonide systemic exposure was similar between age groups when BOS volume and dose adjustments were made for younger (2–9 years old) and older (10–18 years old) children with EoE.
- Volume and dose adjustments to account for age and a shorter esophageal length in younger children (less than 10 years old) are thus appropriate for the treatment of pediatric patients with EoE.

Eosinophilic esophagitis (EoE) is a chronic immune-/allergen–mediated disease characterized by eosinophilic infiltration of the esophageal mucosa (≥15 eosinophils per high-power field [eos/hpf]) and esophageal dysfunction (1,2).

Common signs and symptoms in pediatric patients include vomiting, abdominal pain, reflux-like symptoms, food refusal, and failure to thrive (2). There is currently no US Food and Drug Administration-approved swallowed topical corticosteroid for EoE (3). Clinical guidelines recommend topical corticosteroids and offer conditional recommendations for proton-pump inhibitors (PPIs) and/or dietary modification over no treatment (4). For pediatric patients, first-line pharmacologic options include PPIs and topical corticosteroids (5).

Budesonide oral suspension (BOS) is a swallowed, viscous, immediate-release topical corticosteroid developed for EoE and optimized to maximize mucosal contact at the esophageal surface (6,7). The efficacy and safety of BOS in EoE have been described in two phase 2 and two phase 3, randomized, placebo-controlled trials (6–9). To date, pharmacokinetic (PK) data for BOS have only been reported in healthy adults and in a population PK analysis in children and adults with EoE and healthy adult volunteers (10,11).

MPI 101-01/NCT00762073 was a phase 2 trial in pediatric patients with EoE; patients received low, medium or high doses of BOS, or placebo (6). Significantly more patients treated with medium or high doses of BOS than placebo experienced improvements in histologic and combined (histologic and symptom) outcomes (6). However, changes in EoE clinical symptom scores (CSS) were similar for BOS- and placebo-treated patients (6). We report the systemic PK profile of BOS from MPI 101-01.

## METHODS

### Study Design and Population

This phase 2, randomized, double-blind, placebo-controlled, dose-ranging study was conducted in patients 2–18 years old with EoE across 16 sites in the United States of America from January 2009 to April 2010 (MPI 101-01/NCT00762073) (6). This study was approved by the Institutional Review Board at each center and conducted in accordance with the International Council for Harmonisation of Good Clinical Practice guidelines and the principles of the Declaration of Helsinki.

Patients with symptoms of esophageal dysfunction (EoE CSS) and histologic evidence of EoE (≥ 20 eos/hpf) were eligible (Supplemental Digital Content 1 details further eligibility criteria, http://links.lww.com/MPG/C840) (6). Additional inclusion/exclusion criteria are presented elsewhere (6).

After a 4-week screening period, 81 eligible patients were randomized (1:1:1:1) to low-, medium- or high-dose BOS, or placebo. Patients in the low- and medium-dose BOS groups received placebo in the morning and BOS in the evening [low-dose: 0.35 mg or 0.5 mg; medium-dose: 1.4 mg or 2.0 mg (2–9 or 10–18 years old, respectively)]; patients in the high-dose BOS group received BOS twice-daily [1.4 mg or 2.0 mg (2–9 or 10–18 years old, respectively)]; placebo-treated patients received placebo twice-daily (Supplemental Digital Content 2, http://links.lww.com/MPG/C840) (6). Adjustments in volume [7 mL (2–9 years old); 10 mL (10–18 years old)] and dose were made to account for age and shorter esophageal lengths in younger versus older children (Supplemental Digital Content 2, http://links.lww.com/MPG/C840). After 12 weeks, patients began a 3-week taper period; patients received treatment once-daily (morning) during week 1 and doses were reduced by 50% during weeks 2 and 3 (6).

### PK Sample Collection and Data Analyses

PK analyses were undertaken for BOS-treated patients who had sufficient blood samples to calculate PK parameters. Patients fasted overnight and delayed their morning dose until instructed to take it at the study site. Patients in the low- and medium-dose groups reversed their usual regimen to receive BOS in the morning and placebo in the evening to enable PK sampling at either the second, fourth, eighth, or twelfth week of therapy. Serial blood samples were taken pre-dose and at 0.5, 1, 2, 3, 4, 6, and 8 hours post-dose. Blood samples were combined with heparin, processed by centrifugation (1500 g for 10 minutes) to collect the plasma, and frozen at −80°C. Plasma samples were processed using solid-phase extraction and analyzed using liquid chromatography-mass spectrometry. Budesonide plasma concentration was assessed using a validated bioanalytical method. The lower limit of quantification for detecting blood plasma levels of budesonide was ~20 pg/mL (0.2 mL sample).

PK parameters were calculated using non-compartmental methods, based on actual sample collection times. Prespecified PK parameters included area under the plasma concentration-time curve from time zero to the last quantifiable concentration (AUC_0–last_); maximum observed plasma concentration (C_max_); time to C_max_ (T_max_); and terminal elimination half-life (T_1/2_). *Post hoc* PK parameters included area under the plasma concentration-time curve during a dosing interval, where tau is 12 or 24 hours for twice-daily or once-daily BOS dosing, respectively (AUC_0–tau_); apparent oral clearance (CL/F); apparent volume of distribution associated with the terminal slope (V_Z_/F); geometric least-squares means [95% confidence interval (CI)] for AUC_0–last_ and C_max_; and ratios of geometric least-squares means (90% CI) for high- versus low-dose BOS groups. Relationships between CL/F and V_Z_/F versus bodyweight and body mass index (BMI) were assessed.

PK assessments were measured for morning doses; therefore, patients in the medium- and high-dose groups received the same dose (Supplemental Digital Content 2, http://links.lww.com/MPG/C840); PK data for these groups were combined and are referred to as the high-dose group.

### Safety Assessments

Safety outcomes have been previously reported (6) and included adverse events (AE); physical examinations; electrocardiograms; vital signs; height and bodyweight measurements; and clinical laboratory tests (chemistry, hematology, urinalysis, serum pregnancy, and morning serum cortisol levels) at prespecified visits throughout the study. Safety assessments for the PK analysis set were determined *post hoc*.

Supplemental Digital Content 1, http://links.lww.com/MPG/C840 details the statistical analyses.

## RESULTS

### Baseline Demographics

Thirty-seven BOS-treated patients had sufficient serum samples for PK analysis (Supplemental Digital Content 3, http://links.lww.com/MPG/C840). The mean (standard deviation) age was 9.6 (4.9) years; most patients were male (83.8%) and white (100%). There were 19 patients 2–9 years old and 18 patients 10–18 years old. Baseline demographics were similar between the low- (n = 9), medium- (n = 15), and high-dose (n = 13) BOS groups (Supplemental Digital Content 3, http://links.lww.com/MPG/C840).

### PK Analyses

**FIGURE 1.**
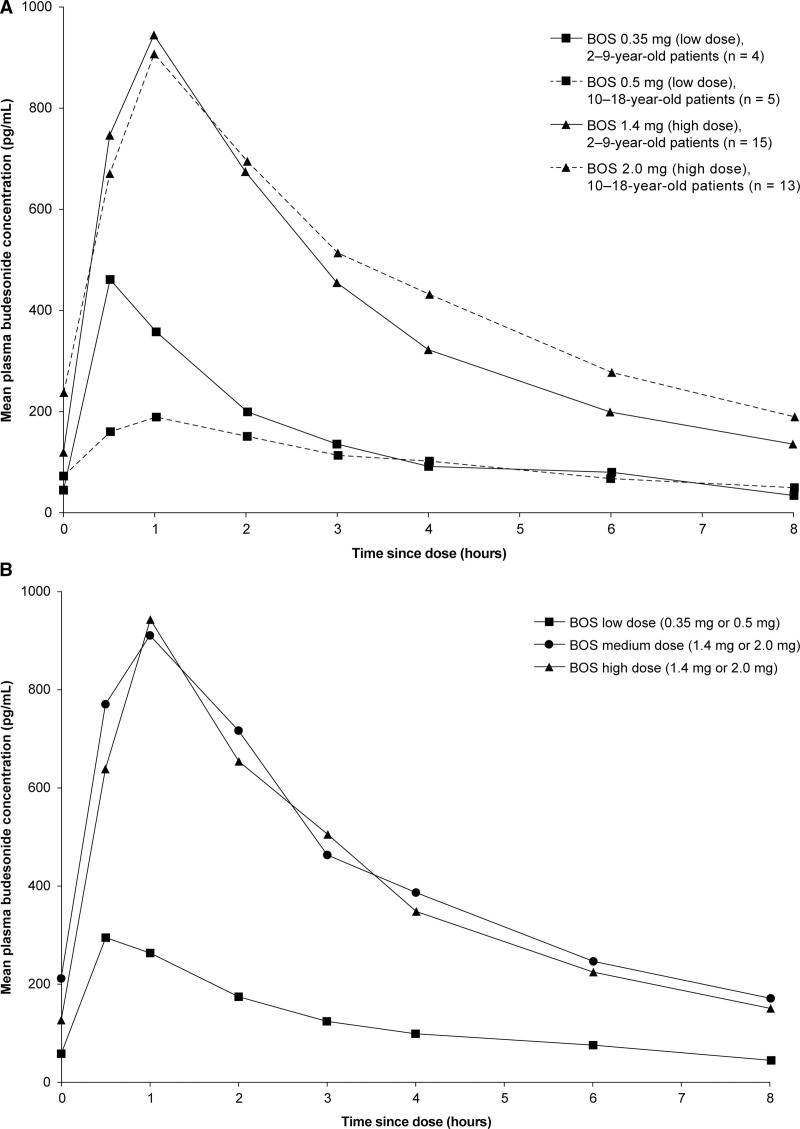
Mean plasma concentrations of budesonide over 8 hours in patients 2–9 and 10–18 years old after an oral dose of BOS (PK analysis set) stratified by (A) age and low- or high-dose and (B) by low-, medium-, or high-dose. BOS = budesonide oral suspension; PK = pharmacokinetic.

Mean budesonide plasma concentrations over time are shown in Figure 1; data were similar for patients in the medium- and high-dose groups, who received the same morning dose of BOS [1.4 mg or 2.0 mg (2–9 or 10–18 years old, respectively)], supporting the combination of these groups for the PK analyses. Drug exposure was consistent between age groups (Fig. 1 and Table 1) and T_max_ was ~1 hour after dosing (Table 1). Mean AUC_0–last_, AUC_0–tau_, and C_max_ increased from the low- to the high-dose group (Table 1). Mean T_max_, CL/F, and V_Z_/F were similar between patients treated with low and high doses of BOS and between age groups (Table 1); minimal variation was observed between treatment groups in T_1/2_, which ranged from 3.3 to 3.5 hours. There was no statistically significant correlation between CL/F and V_Z_/F, and bodyweight or BMI (Supplemental Digital Content 4, http://links.lww.com/MPG/C840 and Supplemental Digital Content 5, http://links.lww.com/MPG/C840). Systemic PK profiles were similar across age strata for each dose; however, for patients 2–9 years old, PK data were only available for four patients treated with low-dose BOS (0.35 mg) (Fig. 1 and Table 1). In this group, one patient (3 years old) had a C_max_ (1060 pg/mL) 2–4 times greater than the other patients, leading to a disparity between the 2 low-dose BOS age groups (2–9 and 10–18 years old).

**TABLE 1.** Summary of prespecified and *post hoc* budesonide PK parameters (PK analysis set) after an oral dose of BOS (low or high dose)

Parameter	Low-dose BOS		High-dose BOS		Ratio of geometric LS means (90% CI) for high- vs low-dose BOS	
	0.35 mg	0.5 mg	1.4 mg	2.0 mg	2–9	10–18
	(2–9 years old)	(10–18 years old)	(2–9 years old)	(10–18 years old)	years old	years old
	(n = 4)	(n = 5)	(n = 15)	(n = 13)		
AUC_0–last_, hour×pg/mL						
Mean	1139.5	743.8	3259.3	3636.9		
SD	800.8	425.3	2109.4	1769.9		
Geometric LS mean	903.9	654.5	2636.4	3282.7	2.92 (1.4–6.2)	5.02 (3.2–7.9)
95% CI	401.8–2033.4	410.9–1042.6	1734.6–4007.1	2459.5–4381.5		
Median	1026.0	551.0	2580.0	2700.0		
Min, max	286, 2220	328, 1390	320, 8270	1730, 7240		
AUC_0–tau_, hour×pg/mL*						
Mean	1710	1270	4260	4840		
SD	846	713	2380	2580		
Median	1780	1050	3150	3640		
Min, max	613, 2670	726, 2230	1750, 10,100	2460, 10,100		
C_max_, pg/mL						
Mean	492.0	195.0	1019.5	958.4		
SD	417.8	64.4	670.2	527.6		
Geometric LS mean	355.2	187.2	805.2	841.1	2.27 (1.0–5.2)	4.49 (2.9–7.0)
95% CI	143.9–877.0	118.4–296.1	504.9–1284.0	633.0–1117.7		
Median	402.0	191.0	812.0	708.0		
Min, max	104, 1060	139, 296	70, 2550	359, 2210		
T_max_, hour						
Mean	0.7	1.2	0.9	1.1		
SD	0.4	0.4	0.4	0.5		
Median	0.5	1.0	1.0	1.0		
Min, max	0.5, 1.2	1, 2	0.5, 2	0.5, 2		
T_1/2_, hour						
Mean	3.3	3.4	3.5	3.5		
SD	0.8	0.8	2.7	1.0		
Median	3.2	3.5	2.7	3.6		
Min, max	2.4, 4.4	2.3, 4.2	1.9, 12.7	2.1, 5.1		
CL/F, L/hour*						
Mean	274	491	417	516		
SD	200	235	192	219		
Median	198	525	446	550		
Min, max	131, 571	224, 689	139, 800	199, 814		
V_Z_/F, L*						
Mean	1300	2240	1860	2570		
SD	888	856	1220	1200		
Median	1110	2110	1760	2660		
Min, max	447, 2520	1360, 3380	496, 5490	1030, 4460		

AUC_0–last_ = area under the plasma concentration-time curve from time zero (T_0_) to the last quantifiable concentration; AUC_0–tau_ = area under the plasma concentration-time curve during a dosing interval, where tau is 12 hours for twice-daily BOS dosing and 24 hours for once-daily BOS dosing; BOS = budesonide oral suspension; CI = confidence interval; CL/F = apparent oral clearance; C_max_ = maximum observed plasma concentration; LS = least-squares; max = maximum; min = minimum; PK = pharmacokinetic; SD = standard deviation; T_1/2_ = terminal elimination half-life; T_max_ = time to C_max_; V_Z_/F = apparent volume of distribution associated with the terminal slope.

* 0.5 mg, n = 4; 1.4 mg, n = 14.

### Safety Assessments

Safety data from this study (N = 81) have been published (6) and demonstrated that all BOS doses were well tolerated. As determined *post hoc*, 75.7% (28/37) of patients from the PK analysis set reported at least one treatment-emergent adverse event (TEAE) over 12 weeks, the proportion of which was higher in patients who received medium- and high-dose BOS (80.0% and 84.6%, respectively) than low-dose BOS (55.6%). However, most TEAEs (83.8%) reported in patients from the PK analysis set were considered by the investigator to be unrelated to study drug; no severe TEAEs or serious AEs were reported, and no TEAEs resulted in study discontinuation. There were no clinically meaningful or dose-related changes in morning cortisol levels, or clinically important changes in other laboratory parameters.

## DISCUSSION

This study determined the systemic PK profile of budesonide for different doses of BOS in patients 2–18 years old with EoE. Systemic exposure for low- and high-dose BOS was similar between age groups. Mean AUC_0–last_, AUC_0–tau_, and C_max_ increased with dose and were consistent between age groups for low- and high-dose BOS. Mean T_max_ and T_1/2_ were similar across age groups and doses. No correlation was observed between CL/F and V_Z_/F, and bodyweight or BMI. The lack of apparent association between these parameters suggests that bodyweight and BMI do not affect the PK profile of BOS. The previously reported efficacy and safety outcomes from MPI 101-01 informed other clinical trial protocols and led to the selection of BOS 2.0 mg twice-daily for further investigation in subsequent trials in adults and adolescents with EoE (6–9).

During our study, BOS was well tolerated and no clinically meaningful changes in morning cortisol levels were recorded (6). More patients who received medium- and high-dose BOS experienced TEAEs than those receiving low-dose BOS; however, most TEAEs were considered unrelated to study drug. A phase 3 study of BOS 2.0 mg twice-daily showed that adrenal effects were infrequently reported with long-term treatment (9).

The mean T_1/2_ of BOS (3.3 to 3.5 hours) indicates that accumulation is not expected with once-daily or twice-daily dosing. The short half-life of BOS is consistent with data on BOS 2.0 mg in healthy adults (10).

The results of one outlier in this study caused disparity between the two age groups; despite this, systemic drug exposure was generally consistent across age strata. Thus, the volume adjustments in this study were a satisfactory means of altering dosing to account for age and esophageal length. It has been reported that esophageal length is correlated with height (12); therefore, the age of 10 years was determined as the cutoff for age stratification, based on the association between puberty onset (typically at 10 years old) and an increase in height (12–14).

The systemic PK of BOS 2.0 mg has been evaluated in 47 adults with EoE (7,15). The geometric means for AUC_0–tau_ [5071 hour×pg/mL (n = 24)] and C_max_ (914.8 pg/mL) in these patients were similar to the mean AUC_0–tau_ (4840 hour×pg/mL) and C_max_ (958.4 pg/mL) in patients 10–18 years old in our study who received BOS 2.0 mg, indicating that volume and dose adjustments used in our study to account for age and esophageal length were appropriate. However, T_max_ was slightly later (~2 hours) than in our pediatric population (~1 hour) (15).

Entocort [Enteric Coated (EC), 9.0 mg budesonide once-daily oral administration] has been evaluated in patients 9–14 years old with Crohn’s disease (16). Systemic exposure to budesonide was higher with Entocort EC than for our patients 10–18 years old who received high-dose BOS (Entocort EC vs BOS 2.0 mg; AUC from time 0 to 24 hours, 17.78 vs 9.68 hour×ng/mL; C_max_, 2.58 vs 0.96 ng/mL), suggesting that BOS 2.0 mg twice-daily may have an improved safety profile over Entocort EC 9.0 mg once-daily (16).

This study was limited by the small population size; one outlier in the low-dose 2–9-year-old BOS group inflated the mean C_max_ for this group. Moreover, only the linear association between PK parameters and bodyweight or BMI was examined. Future dose-ranging studies of swallowed topical corticosteroids could consider alternative methods for volume and dose adjustments, such as using body surface area.

## Conclusions

Overall, PK parameters were similar across age strata, suggesting that volume and dose adjustments were a satisfactory means of adjusting the BOS dose to account for age and esophageal length in pediatric patients. Additionally, the mean half-life of BOS indicated that accumulation is not expected with the dosing regimens utilized in this study.

These findings support an ongoing robust clinical development program of this topical corticosteroid optimized for esophageal delivery and will inform dose adjustments for patients younger than 11 years old for future studies of BOS.

## Acknowledgments:

The authors would like to thank Elaine Phillips, PhD, for her contribution to this study. The authors also gratefully acknowledge the investigators, study coordinators, and patients and their families for their participation in this study.

## Supplementary Material


