# Supplementary material for: Pharmacokinetics of Budesonide Oral Suspension in Children and Adolescents With Eosinophilic Esophagitis
Source: J Pediatr Gastroenterol Nutr. 2022 Jun 6;75(2):186–91. doi: 10.1097/MPG.0000000000003482 (PMC9278710; doi:10.1097/MPG.0000000000003482)
Supplement: Supplementary file 1 [file mpg-75-186-s001.pdf]

Supplemental Digital Content 1

Supplemental Methods

Key Inclusion and Exclusion Criteria

Proton-pump inhibitor (PPI) use was permitted providing patients had been on a stable dose regimen in the 4 weeks before the study-qualifying esophageal biopsy; patients continued their treatment regimen throughout the study. Patients with a morning cortisol level below the lower limit of normal (per the study laboratory age- and sex-specific reference range [0.6–5.0 µg/dL]) during the screening period were excluded from study participation.

Compliance with the study drug, which was evaluated at each study visit, was required to continue study participation.

Inhaled or topical corticosteroids for conditions other than eosinophilic esophagitis (EoE), such as asthma or allergic rhinitis, were permitted during the study if patients had been on a stable dose regimen in the 4 weeks before the study-qualifying esophageal biopsy and would continue this regimen throughout the study; however, other swallowed topical corticosteroids for EoE and systemic (oral or parenteral) corticosteroids were prohibited (1).

Study Drug Administration

Budesonide oral suspension (BOS) was supplied in multi-dose, amber glass bottles (with a graduated medicine dropper or graduated teaspoon), each containing 120 mL of BOS with budesonide concentrations of either 0.2 mg/mL, 0.05 mg/mL or 0.00 mg/mL (for placebo). Study medication was stored at room temperature, with care taken not to exceed 77°F (25°C), with the cap closed and the bottle protected from the light.

Pharmacokinetic Sample Collection

Pharmacokinetic (PK) blood sampling occurred at one study visit at the discretion of the study site. For patients 2–9 years old in the low-dose BOS group, sampling occurred at week 4 (n = 1), week 8 (n = 1), and week 12 (n = 2). For patients 10–18 years old in the low-dose BOS group, sampling occurred at week 4 (n = 1), week 8 (n = 3), and week 12 (n = 1). For patients 2–9 years old in the medium-dose BOS group, sampling occurred at week 4 (n = 2), week 8 (n = 3), and week 12 (n = 2). For patients 10–18 years old in the medium-dose BOS group, sampling occurred at week 4 (n = 3), week 8 (n = 3), and week 12 (n = 2). For patients 2–9 years old in the high-dose BOS group, sampling occurred at week 2 (n = 2),

week 4 (n = 1), and week 12 (n = 5). For patients 10–18 years old in the high-dose BOS group, sampling occurred at week 4 (n = 2), week 8 (n = 1), and week 12 (n = 2).

Statistical Analyses

Pre-specified parameters including baseline demographics and characteristics, budesonide plasma concentrations, and PK parameters were summarized for each age group and within each BOS treatment group (low-, medium-, and high-dose) for patients in the PK analysis set using descriptive statistics, including mean (standard deviation), number (%), and median (minimum, maximum). Relationships between apparent oral clearance (CL/F) and bodyweight, CL/F and body mass index (BMI), apparent volume of distribution (V_Z/F) and bodyweight, and V_Z/F and BMI were explored graphically *post hoc*; a linear model was fit to CL/F and V_Z/F versus BMI and to V_Z/F versus bodyweight to examine the statistical significance ($p \leq 0.05$) of any relationship between these parameters. Similarly, a *post hoc* allometric equation was used to examine the relationship between CL/F and bodyweight. The mean bodyweight during this study was 39.25 kg; therefore, the allometric equation was centered on 40 kg to obtain the following equation:

$$\frac{CL}{F} = \frac{CL_{TV}}{F} * \left(\frac{BW}{40}\right)^{\alpha}$$

CL_{TV}/F = CL/F for a patient with a typical bodyweight value of 40 kg.

TV = typical value

α = the allometric exponent.

Estimation of the allometric exponent was enabled by fitting the equation to the data and to a fixed α value of 0.75.

Reference

1 Gupta SK, Vitanza JM, Collins MH. Efficacy and safety of oral budesonide suspension in pediatric patients with eosinophilic esophagitis. *Clin Gastroenterol Hepatol* 2015;13:66–76.

Supplemental Digital Content 2

Table. Study treatment by BOS dose and age group (PK analysis set, N = 37)

BOS dose group	Age group, years	n	BOS volume, mL	Budesonide concentration, mg/mL	Morning budesonide dose, mg	Evening budesonide dose, mg	Total budesonide dose, mg/day
**Low**	2–9	4	7	0.05	Placebo	0.35	0.35
	10–18	5	10			0.5	0.5
**Medium**	2–9	7	7	0.2	Placebo	1.4	1.4
	10–18	8	10			2.0	2.0
**High**	2–9	8	7	0.2	1.4	1.4	2.8
	10–18	5	10		2.0	2.0	4.0

Volume adjustments were also made for placebo to maintain double-blind dosing. Patients in the high-dose BOS groups received the active study drug twice daily; therefore, the total daily doses were 2.8 mg and 4.0 mg for patients 2–9 and 10–18 years old, respectively (1). However, for the purposes of the systemic PK analyses, the PK assessments were carried out after morning doses of BOS, at which point the medium- and high-dose groups received the same dose of BOS. BOS = budesonide oral suspension; PK = pharmacokinetic.

Reference

1 Gupta SK, Vitanza JM, Collins MH Efficacy and safety of oral budesonide suspension in pediatric patients with eosinophilic esophagitis. Clin Gastroenterol Hepatol 2015;13:66–76.

Supplemental Digital Content 3

Table. Baseline demographics and patient characteristics (PK analysis set, N = 37)

**Demographic/characteristic**	**Low-dose BOS (n = 9)**	**Medium-dose BOS (n = 15)**	**High-dose BOS (n = 13)**	**Overall (N = 37)**
**Age, years, mean (SD)**	10.7 (5.8)	10.6 (4.9)	7.8 (3.7)	9.6 (4.9)
**Age group, n (%)**				
2–9 years old	4 (44.4)	7 (46.7)	8 (61.6)	19 (51.3)
10–18 years old	5 (55.6)	8 (53.3)	5 (38.4)	18 (48.7)
**Male, n (%)**	7 (77.8)	13 (86.7)	11 (84.6)	31 (83.8)
**Race, n (%)**				
White	9 (100.0)	15 (100.0)	13 (100.0)	37 (100.0)
**Ethnicity, n (%)**				
Hispanic or Latino	0 (0.0)	0 (0.0)	1 (7.7)	1 (2.7)
Not Hispanic or Latino	9 (100.0)	15 (100.0)	12 (92.3)	36 (97.3)
**Time since diagnosis of EoE, months, mean (SD)**	16.8 (17.4)	32.0 (36.6)	16.6 (23.2)	22.9 (28.7)
**Height, cm, mean (SD)**	141.2 (30.6)	145.8 (27.5)	132.3 (26.8)	140.0 (27.9)
**Weight, kg, mean (SD)**	42.0 (23.5)	46.3 (27.9)	33.4 (20.5)	40.7 (24.4)

Data for patients treated with low-, medium-, and high-dose BOS are presented for patients 2–18 years old (2–9- and 10–18-year-old groups combined). BOS = budesonide oral suspension; EoE = eosinophilic esophagitis; PK = pharmacokinetic; SD = standard deviation.

Supplemental Digital Content 4

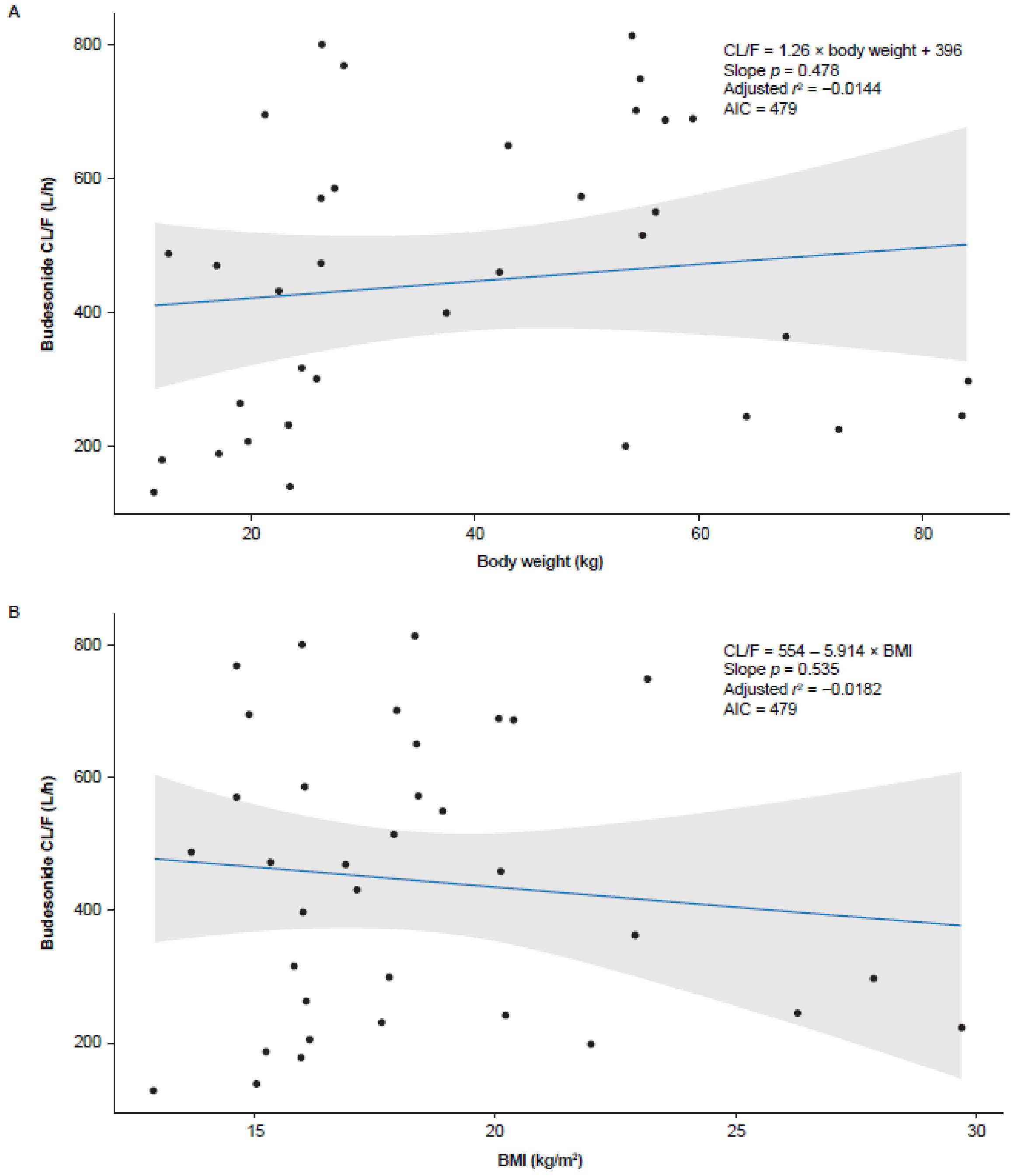

Figure. Fit of a linear model to assess the relationship between budesonide CL/F and (**A**) bodyweight and (**B**) BMI (PK analysis set, N = 37). AIC = Akaike information criterion; BMI = body mass index; CL/F = apparent oral clearance.

Supplemental Digital Content 5

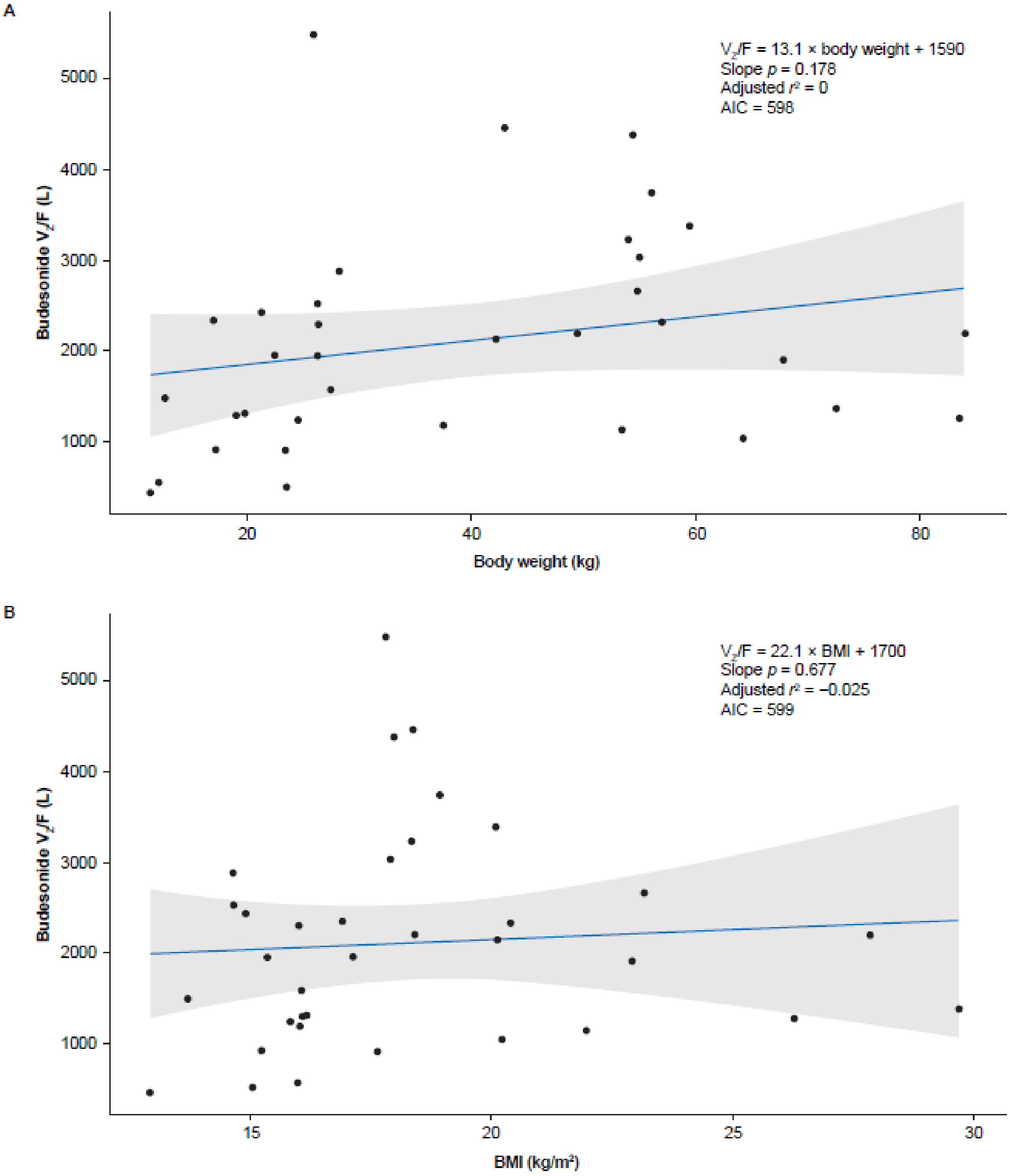

Figure. Fit of a linear model to assess the relationship between budesonide V_Z/F and (**A**) bodyweight and (**B**) BMI (PK analysis set, N = 37). AIC = Akaike information criterion; BMI = body mass index; V_Z/F = apparent volume of distribution associated with the terminal slope.
